# Evaluation of the District Health Information System in District Kotli, Azad Jammu and Kashmir: A Retrospective Analysis

**DOI:** 10.7759/cureus.53242

**Published:** 2024-01-30

**Authors:** Mohammad Saleem Khan, Khawaja Faizan Ejaz, Khan Adnan, Sohail Ahmed, Humayun Saleem, Sarosh Khan Jadoon, Amna Akbar, Sabahat Tasneem

**Affiliations:** 1 Medicine, District Headquarters Hospital, Kotli, PAK; 2 Internal Medicine, Russells Hall Hospital, Dudley, GBR; 3 Gastrointestinal Surgery, Yangtze University, Jingzhou, CHN; 4 Public Health, Health Services Academy, Islamabad, PAK; 5 General Surgery, Combined Military Hospital, Muzaffarabad, PAK; 6 Emergency and Accident, District Headquarters Hospital, Muzaffarabad, PAK

**Keywords:** time-series data management, non-communicable diseases, communicable diseases, global burden of disease, district healthcare

## Abstract

Background: It is essential to implement a high-quality electronic database for keeping important information. The District Health Information System (DHIS) is an active data-keeping system in Pakistan. This study aimed to evaluate the patients' data from the DHIS dashboard for the District Headquarters Hospital, Kotli, Azad Jammu and Kashmir (AJK).

Methodology: The data was requested from the hospital administration at District Headquarters Hospital, Kotli, AJK, and the data was analyzed after permission was granted. The data was given in two forms; one was a hard copy of the data for August and September and the other was a comma-separated values file for October and November, 2023.

Results: The highest frequency of patients was received in the department of emergency and trauma and the patient's median age was between 15 and 49 years. The second department was medicine with the >50 years of age. Common conditions that needed more attention were chronic obstructive pulmonary disease, acute respiratory infection, diarrhea, pneumonia, diabetes mellitus, hypertension, and ischemic heart disease.

Conclusion: For nations with constrained healthcare systems and funds, primary health care (PHC) is the only viable approach for managing non-communicable diseases (NCDs). However, PHC systems intended for infectious diseases have not sufficiently adapted to the growing requirement of chronic care for NCD. Research using health information databases offers numerous benefits, such as the evaluation of large data sets and unexpected prevalence of disease in certain populations, such as a higher prevalence of disease in one gender or age group. Health information system-based data analysis or studies are less expensive and faster but lack scientific control over data collection.

## Introduction

Every healthcare system should be able to comprehend the level of care it provides, and consumers should know the quality of care they receive. To meet these demands, both quantitative and qualitative data analysis are essential. The data are used to answer questions related to quality of service, monitor, and facilitate improvement in healthcare, as well as to make recommendations and decisions. We can use the same data in different ways, depending on the type of question we are looking for. The analysis of both quantitative and qualitative data is important for assessing and directing the process of change. Time series analysis is the gold standard for using data for improvement over intervals of time, which requires frequent, small-scale data collection and presentation efforts [1].

The secure and effective transfer of private health information is ensured by placing reliable data management systems in place. Healthcare professionals usually overlook the responsibility of keeping meticulous data and clinical records of patients. Therefore, it is critically important to implement high-quality electronic healthcare record applications that can reduce errors caused by poor data-keeping. Medical errors are typically caused by inadequate systems for processing medical data [2]. District Health Information Systems (DHIS) became active in Pakistan in 2008. It has been functional since 2009 in Punjab and other provinces. It was established with the aim of improving data quality and analyzing it to encourage evidence-based decision-making [3].

DHIS is monitored, and its data are analyzed for policy guidance by the Ministry of National Health Services Regulation and Coordination. DHIS is now digitalized to promote data flow through a management dashboard. The Pakistan Health Information System dashboard is established at the national level and housed in the Health Planning, System Strengthening, and Information Analysis Unit [4].

Managing pharmacy stock and human resource management, maintaining hospital records, infection control, and data monitoring are some of the functions of health management systems (HMSs). HMSs can help improve health delivery, for example, skilled birth attendance in China was improved, and antenatal and postnatal care was enhanced in Egypt with the help of HMS [5]. DHIS was established in Pakistan with the same vision. The objective of our study was to emphasize the importance of a robust mechanism of data management at healthcare centers. A system that keeps patients' records can help to determine the burden on healthcare and evaluate the performance. The study evaluated the DHIS in phase one; both in the form of paper (hard copy) and a digital dashboard. We aimed to determine the efficacy of keeping data when the patients' records are kept in the form of paper or a digital system like DHIS.

## Materials and methods

Purpose

The study of this data will give an estimate of the burden of patients on the hospital and its capacity to accommodate the patient flow. For this purpose, we will retrospectively determine the number of services gained in the hospital by patients and the number of visitors in each department. It will also emphasize on importance of a robust data-keeping system.

Study setting

Kotli is a district administrative unit in Azad Jammu and Kashmir (AJK) covering an area of 1862 square kilometers with a population of 0.828 million (projected population 2021) which accounts for 19% of the total population of AJK. With a growth rate of 1.69, the population comprises 391,465 men and 436,580 women. There are more than 250 healthcare facilities in the district including basic health units (BHUs) and dispensaries. The burden of patients on healthcare facilities is 2977 patients per facility. The District Headquarters Hospital, Kotli is the only tertiary care facility, so it caters to the majority of first-visit patients in outpatient departments (OPDs) and patients referred from rural health centers, BHUs, and dispensaries [6].

Data

The data set that we are using is the time series data created as the baseline for the DHIS. The permission to access the data was obtained from the concerned authorities before data collection. The DHIS was introduced in Pakistan in 2007-2008 and is functional since 2009-2010. The DHIS in AJK became functional quite late but it is in use now. The system was introduced recently in District Headquarters Kotli and it is in the first phase or a trial phase. The trial phase comprised of keeping data in two forms; first in the form of paper (hard copy) for August and September and the second as a digital copy on the dashboard of DHIS for October and November 2023.

Data collection

The data was provided in the form of hard copies (paper sheets) for August and September while it was given in the form of a comma-separated values (CSV) file on a portable device for October and November (no direct access to the dashboard). For data collection, the relevant information was searched on the official documents provided by the hospital. The record contains data for outpatients of each department including medicine, surgery, pediatrics, dermatology, dentistry, eye, ear, nose throat (ENT), and orthopedics, etc. The details of infectious diseases and communicable diseases like malaria, dengue, tuberculosis (TB), and hepatitis B and C are given separately in the documents. The data is presented in the form of outpatients’ department visits (number of patients) and the number of patients attended at each department. The main OPD deals with the segregation of patients into each department. The data is distributed in different ways; the first distribution is in departments; and the second distribution is outdoor and indoor patients. The third distribution is the type of disease diagnosed and the fourth is the distribution on the basis of age groups. The first age group was under one year, the second group was one to four years, and the third group was above five years. About 15 to 49 years is the fourth group and then the 50 years and older age group was the last one. As this was a baseline data entry for DHIS, two methods of data entry were used; one was data entry on hard copy as phase one and the second was data entry on the DHIS dashboard. The data was recorded from hard copies for August and September on an Excel sheet and for October and November was copied in the form of a CSV file and then transported onto an Excel sheet.

Data analysis

The number of patients depicted as the frequency of visits in each department was noted down and stratified against age groups. The maternal, newborn, and child health (MNCH) visits and family planning (FP) visits were evaluated separately. The analysis was done on an Excel sheet as the study did not aim to determine any correlation or associations. The data was described based on records given on the Excel sheet and the graphs were also made on the Excel sheet. Any necessary analysis like sum and average was performed on the Excel sheet. Due to the simple nature of the analysis, no statistical tool was applied.

## Results

**Figure 1:**
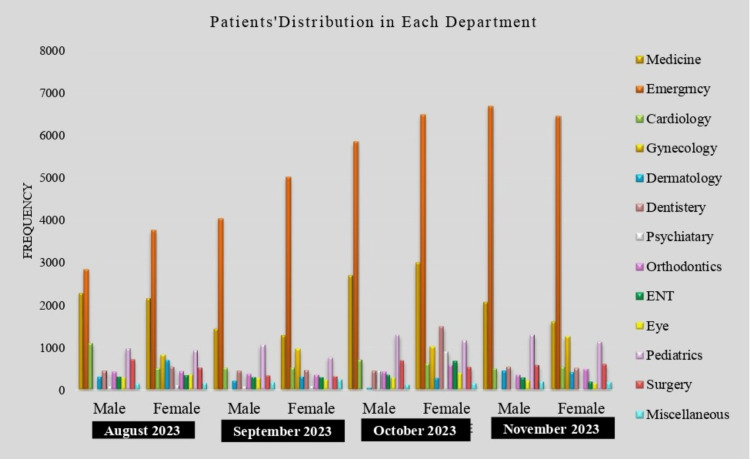
Frequency of patients who attended each department from August 2023 to November 2023 ENT: Ear, nose, throat

The data was taken from the DHIS dashboard for four months (August to November 2023) only as the DHIS dashboard is in the initial phase and limited data is available. The frequency of patients attending each department was variable (Figure 1).

The emergency department received the highest number of patients followed by the department of medicine.

The detailed distribution of patients is given in Table 1. This gives an overall number of the patients who were attended at each department at the hospital. The frequency is then stratified based on the age of the patients and their gender (Table 1).

**Table 1:** Outpatient department attendance in each department at the District Headquarter Hospital ENT: Ear, nose, throat

		August 2023		September 2023		October 2023		November 2023	
	Age	Male	Female	Male	Female	Male	Female	Male	Female
Medicine	<1 year	0	0	0	0	0	0	0	0
	1-4 years	0	0	0	0	0	0	0	0
	5-14 years	0	0	0	0	0	261	0	0
	15-49 years	1739	1620	971	852	1435	1518	1130	846
	50+ years	556	551	490	435	1274	1234	954	786
	Total	2295	2171	1461	1287	2709	3013	2084	1632
Surgery	<1 year	7	0	19	4	23	1	45	22
	1-4 years	164	115	39	12	113	180	150	52
	5-14 years	194	138	87	31	192	200	82	69
	15-49 years	175	178	85	161	244	0	196	188
	50+ years	183	100	123	121	133	172	126	283
	Total	723	531	353	329	705	553	599	614
Pediatrics	<1 year	360	245	303	258	500	325	151	133
	1-4 years	340	447	427	263	272	383	662	511
	5-14 years	283	247	339	259	528	460	491	493
	15-49 years	0	0	0	0	0	0	0	0
	50+ years	0	0	0	0	0	0	0	0
	Total	983	939	1069	780	1300	1168	1304	1137
Eye	<1 year	0	20	9	8	7	5	8	5
	1-4 years	20	12	9	12	14	12	15	2
	5-14 years	16	18	19	7	20	160	13	15
	15-49 years	102	177	80	108	106	70	53	17
	50+ years	168	143	170	113	151	150	132	125
	Total	306	370	287	248	298	397	221	164
ENT	<1 year	15	27	4	8	15	20	14	17
	1-4 years	67	73	49	45	73	90	19	58
	5-14 years	73	76	49	73	71	70	52	16
	15-49 years	45	82	38	78	70	391	80	85
	50+ years	118	101	173	113	136	117	126	22
	Total	318	359	313	317	365	688	291	198
Orthopedics	<1 year	19	11	0	0	10	21	5	20
	1-4 years	134	105	26	31	71	86	29	7
	5-14 years	93	71	16	0	115	391	33	76
	15-49 years	142	110	260	188	163	9	161	237
	50+ years	44	143	78	141	69	90	130	151
	Total	432	440	380	360	428	597	358	491
Psychiatry	<1 year	0	0	0	0	1	0	0	0
	1-4 years	0	0	0	0	6	12	0	0
	5-14 years	0	0	0	0	57	211	0	27
	15-49 years	9	20	13	17	261	400	0	0
	50+ years	75	96	72	78	131	277	0	0
	Total	84	116	85	95	456	900	0	27
Dental	<1 year	1	10	1	2	11	14	0	0
	1-4 years	8	25	3	4	48	95	3	0
	5-14 years	75	155	103	108	50	400	60	0
	15-49 years	284	281	143	98	285	969	270	210
	50+ years	82	72	200	251	52	21	210	304
	Total	450	543	450	463	446	1499	543	514
Dermatology	<1 year	3	18	11	9	0	0	10	30
	1-4 years	31	52	40	28	0	0	56	38
	5-14 years	50	146	99	53	0	9	58	43
	15-49 years	197	430	40	199	6	211	296	293
	50+ years	23	56	22	26	46	60	34	27
	Total	304	702	212	315	52	280	454	431
Gynecology	<1 year	0	0	0	0	0	0	0	0
	1-4 years	0	0	0	0	0	0	0	0
	5-14 years	0	0	0	0	0	29	0	20
	15-49 years	0	635	0	670	0	738	0	960
	50+ years	0	196	0	304	0	267	0	283
	Total	0	831	0	974	0	1034	0	1263
Cardiology	<1 year	1	0	0	0	1	0	0	0
	1-4 years	0	0	0	0	0	0	0	0
	5-14 years	3	28	13	1	35	44	16	42
	15-49 years	273	256	212	330	325	300	139	201
	50+ years	827	235	310	212	362	284	355	312
	Total	1104	519	535	543	723	628	510	555
Emergency	<1 year	17	223	118	340	190	212	201	249
	1-4 years	301	360	230	316	262	454	270	249
	5-14 years	412	425	216	401	433	3612	369	0
	15-49 years	310	430	2469	2810	4051	2210	4111	3969
	50+ years	1807	2331	1013	1156	922	0	1749	1998
	Total	2847	3769	4046	5023	5858	6488	6700	6465
Miscellaneous	<1 year	7	0	8	0	0	1	1	1
	1-4 years	30	3	37	18	33	22	83	15
	5-14 years	1	0	0	0	1	0	0	0
	15-49 years	25	37	33	81	26	49	21	46
	50+ years	93	125	108	155	74	92	103	126
	Total	156	165	186	254	134	164	208	188

The highest frequency of the patients was received at the hospital in October and November in the age group of 15 to 49 years. The attendance of males is higher than female patients.

**Figure 2:**
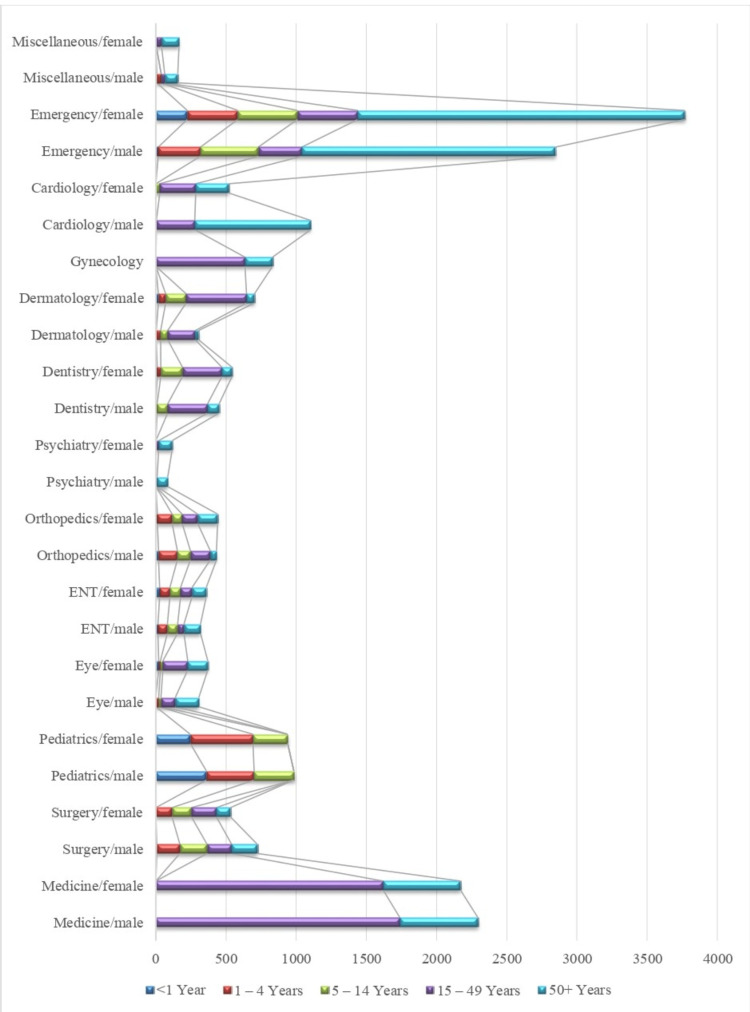
Stratification of patients according to department, gender, and age in August 2023 ENT: Ear, nose, throat

The data is stratified based on gender and age in each department for August (Figure 2).

In August, the highest burden of patients was received by the department of emergency and trauma. Number of female patients of the age ≥50 years (n = 2331) of age surpassed every other age category. The second most burdened department was medicine, and the most affected group was males aged 15 to 49 years (n = 1739).

**Figure 3:**
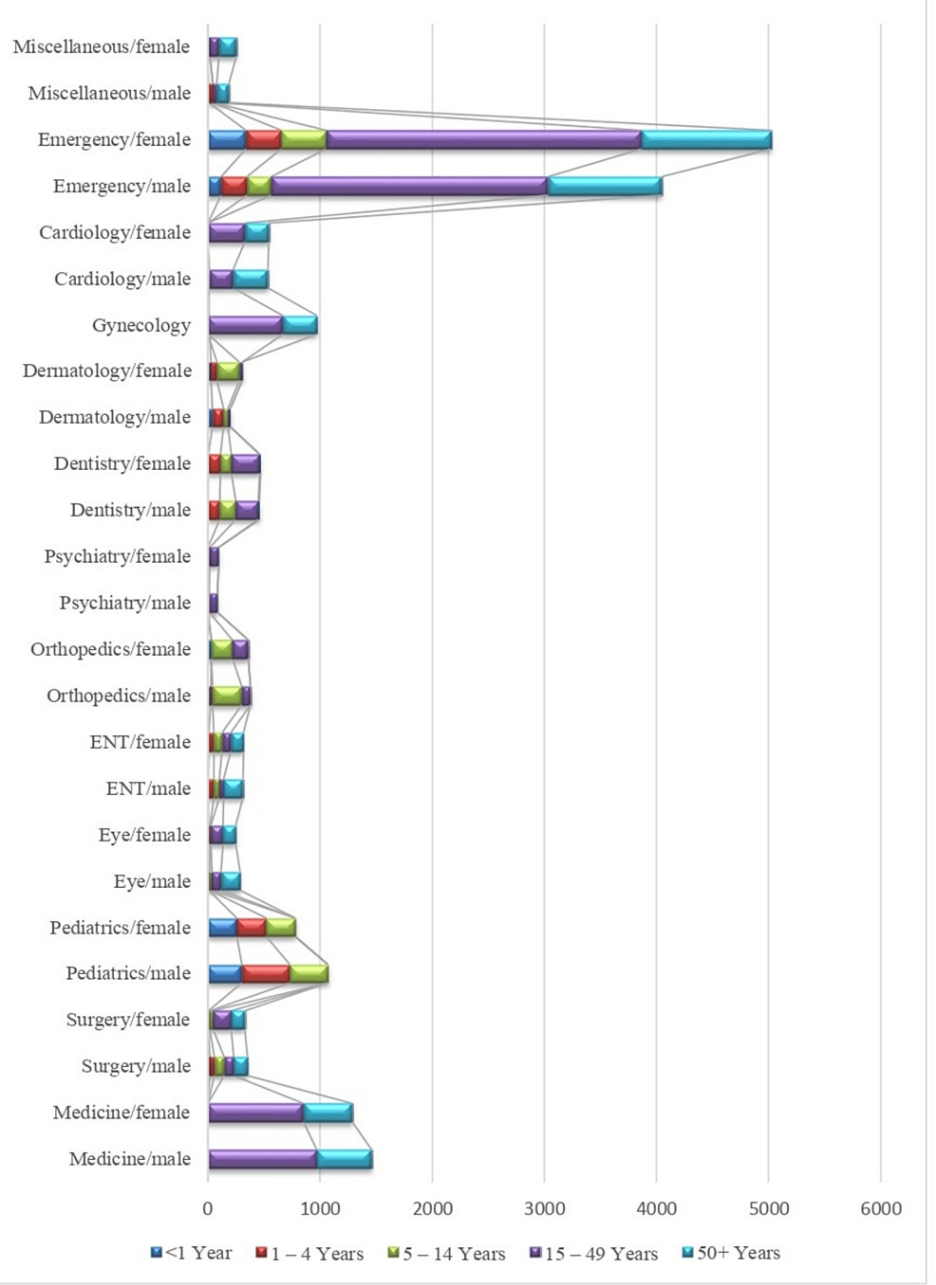
Stratification of patients according to department, gender, and age in September 2023 ENT: Ear, nose, throat

The frequency of patients received in each department is stratified based on gender and age for September (Figure 3).

The hospital received a maximum number of patients in the age group of 15 to 49 years in the department of emergency and trauma. The female patient’s frequency (n = 2810) was more than males.

**Figure 4:**
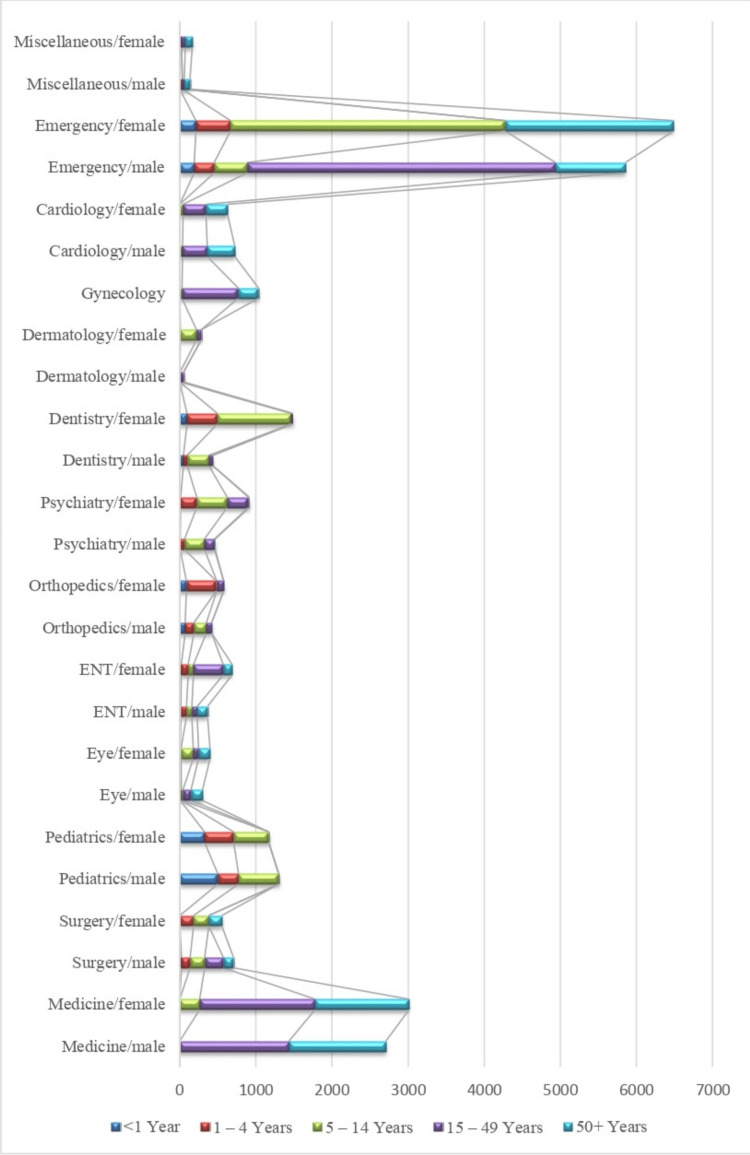
Stratification of patients according to department, gender, and age in October 2023 ENT: Ear, nose, throat

The data reported in every department is stratified based on gender and age for October (Figure 4).

Females of the age group 5 to 14 years presented most in the department of emergency and trauma (n = 3612) after males of the same age group (n = 4051), the second age group of females was ≥50 years (n = 2210).

**Figure 5:**
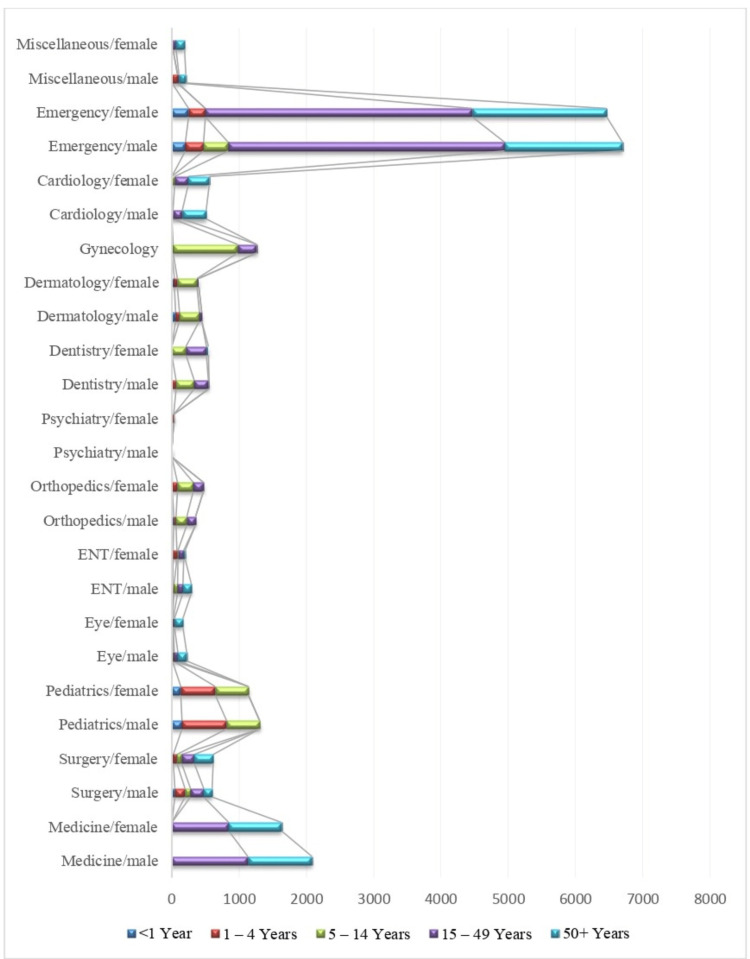
Stratification of patients according to department, gender, and age in November 2023 ENT: Ear, nose, throat

The patient data gathered for each department is stratified based on gender and age for November (Figure 5).

In November, the emergency and trauma department again received the highest number of patients compared to all departments. With males of age group 15 to 49 presenting the highest frequency (n = 4111) and females of age group ≥50 years (n = 1998).

The number of patients diagnosed with a particular disease was noted down in this (Table 2).

**Table 2:** Distribution of patients' frequency based on diagnosis established in each patient ENT: Ear, nose, throat

	Respiratory Diseases	August 2023	September 2023		October 2023		November 2023
1	Acute (upper) respiratory infections (ARI)	1409	841		1600		1684
2	Pneumonia <5 years	144	123		274		352
3	Pneumonia >5 years	182	106		334		472
4	Tuberculosis (TB) suspects	171	133		202		45
5	Chronic obstructive pulmonary diseases	1509	489		1274		1124
6	Asthma	487	455		476		842
Gastrointestinal diseases							
1	Diarrhea/dysentery in <5 years	411	411		518		749
2	Diarrhea/dysentery in >5 years	838	396		553		577
3	Enteric/typhoid fever	20	0		60		50
4	Peptic ulcer diseases	1399	766		1519		853
5	Cirrhosis of liver	34	67		40		62
Urinary tract diseases							
1	Urinary tract infections (UTI)	431	438		519		473
2	Nephritis/nephrosis	35	39		55		53
3	Benign enlargement of the prostate	75	50		45		65
Other communicable diseases							
1	Suspected malaria	137	3		61		10
Vaccine-preventable diseases							
1	Suspected viral hepatitis	41	36		27		46
Cardiovascular diseases							
1	Ischemic heart disease (IHD)	389	365		456		339
2	Hypertension	808	630		751		713
Skin diseases							
1	Scabies	506		200		350	600
2	Dermatitis	260		100		300	285
Endocrine diseases							
1	Diabetes mellitus	731		540		578	777
Neuro-psychiatric diseases							
1	Depression	48		66		15	10
2	Drug dependence	6		6		0	0
3	Epilepsy	12		14		5	2
Eye and ENT							
1	Cataract	150		130		150	120
2	Trachoma	0		30		0	0
3	Glaucoma	15		0		25	30
4	Otitis media	150		220		250	250
Oral diseases							
1	Dental caries	487		681		437	574
Injuries/poisoning							
1	Road traffic accidents	439		515		551	435
2	Fractures	0		129		89	734
3	Burns	12		27		60	43
4	Dog bite	115		45		93	106
5	Snake bites (with signs/symptoms of poisoning)	11		23		2	0
Miscellaneous diseases (surveillance importance)							
1	Acute flaccid paralysis	0		0		0	0
2	Suspected HIV/AIDS	0		0		0	0

The most frequent diseases were acute respiratory infection, peptic ulcer disease, and chronic obstructive pulmonary disease (cases of more than 4000 for each diagnosis). These were followed by diarrhea (both <5 and >5 years), asthma, and hypertension (the number of cases ranged between 2000 and 3000). Then comes diabetes mellitus (DM), ischemic heart disease (IHD), scabies, urinary tract infection, dental caries, and pneumonia (cases ranged between 1000 and 2000). Discussing the diseases that need surveillance, 150 viral hepatitis, 155 tuberculosis, and 211 cases of suspected malaria were reported. No cases of AIDS/HIV or acute flaccid paralysis were reported.

The hospital provides services apart from regular OPD attendance. These are immunization, FP services, maternal and newborn health which includes antenatal visits and mothers' vaccination, tuberculosis-directly observed treatment (TB-DOTS), and birth and neonatal care (Table 3).

**Table 3:** Special services EPI: Expanded Programme on Immunization; TT: Tetanus Toxoid; TB: Tuberculosis; LBW: Low birth weight; FP: Family planning

Immunization (from EPI register)		August 2023	September 2023	October 2023	November 2023
1	Children <12 months received their third pentavalent vaccine	82	80	85	80
2	Children <12 months received their first measles vaccine	90	101	66	76
3	Children <12 months fully immunized	90	101	66	76
4	Pregnant women received TT-2 vaccine	48	49	38	34
Tuberculosis-directly observed treatment (TB-DOTS) (from TB card TB-01)					
1	Intensive-phase TB-DOTS patients	0	19	10	10
Family planning (FP) services/commodities provided (from FP register)					
1	Total FP visits	114	315	304	333
2	Combined oral contraceptives (COC) cycles	31	146	157	170
4	depot medroxyprogesterone acetate (DMPA) injection	30	41	22	27
5	Condom pieces	336	778	44	732
6	Intrauterine contraceptive devices (IUCD)	0	0	2	12
Maternal and newborn health (from maternal health and obstetric registers)					
1	First antenatal care visits (ANC-1) in the facility	291	262	363	440
2	Antenatal care revisit in the facility	0	8	0	0
3	First postnatal care visit (PNC-1) in the facility	0	0	12	15
Deliveries in the facility					
1	Normal vaginal deliveries in the facility	173	148	111	200
2	Cesarean sections	158	151	174	178
3	Live births in the facility	319	242	276	373
4	Live births with LBW (<2.5 kg)	0	113	101	35
5	Stillbirths in the facility	0	7	9	5
Neonatal deaths in the facility					
1	Birth trauma	0	2	0	0
2	Birth asphyxia	12	0	0	5
3	Prematurity	10	12	8	10

The children under 12 months who received their first or third vaccination were 333 and 327, respectively. About 169 women received an anti-tetanus vaccine and 333 children were fully immunized. The hospital facilitated 1066 FP visits, 1356 first antenatal visits, and 632 normal deliveries. The hospital also provides laboratory services (Table 4).

**Table 4:** Details of laboratory services OPD: Outpatient department; MP: Malarial parasite; AFB: Acid-fast bacillus

Service provided					
1	Total lab investigations	August 2023	September 2023	October 2023	November 2023
	OPD	4582	890	4271	4081
	Indoor	21807	18530	15733	28135
2	Total X-rays				
	OPD	1780	2335	2202	1861
	Indoor	341	416	412	424
3	Total ultra sonographies				
	OPD	895	1060	891	903
	Indoor	334	426	305	290
4	Total computed tomography (CT) scans				
	OPD	0	0	0	287
	Indoor	0	0	0	136
5	Total electro cardiograhies (ECGs)				
	OPD	534	554	285	769
	Indoor	302	230	281	318
Laboratory investigation for communicable diseases					
Malaria					
1	Slides examined	137	67	61	10
2	Slides MP +ve	0	1	3	0
3	Slides Plasmodium falciparum +ve	0	0	0	0
Tuberculosis					
1	Slides for AFB Diagnosis	171	216	20	45
2	Diagnosis slides with AFB +ve	7	3	7	3
3	Follow-up slides for AFB	0	0	7	0
4	Follow-up slides with AFB +ve	0	0	0	0
Viral hepatitis and HIV					
1	Patients screened	1786	231	1746	1898
2	Hepatitis B +ve	16	4	8	15
3	Hepatitis C +ve	0	0	0	64
4	HIV +ve	0	0	50	0

The laboratory investigations are done for regular infections as well as for communicable diseases including malaria, tuberculosis, and viral hepatitis.

The patients can be admitted to the hospital as the district headquarters has the facility for indoor admissions and services (Table 5).

**Table 5:** Details of hospital indoor services/burden in each department Details of all the departments' indoor patient burden from August to November. DM: Data missing, LAMA: Left against medical advice; Disc/DOR: Discharge/discharge on request; ALS: Average length of stay; OB/GYN: Obstetrics/gynecology; ENT: Ear, nose, throat

1	Medicine	August 2023	September 2023	October 2023	November 2023
	Allocated beds	50	DM	50	50
	Admissions	524	DM	503	662
	Disc/DOR not on same day	368	DM	334	439
	Disc/DOR on same day	0	DM	0	0
	LAMA	23	DM	21	33
	Referred	47	DM	59	64
	Deaths	31	DM	29	48
	Total daily patient count	1107	DM	1351	1503
	Bed occupancy rate	71%	DM	90%	97%
	ALS	2.4	DM	0	2
2	Surgery				
	Allocated beds	84	DM	84	84
	Admissions	430	DM	430	400
	Disc/DOR not on same day	372	DM	360	276
	Disc/DOR on same day	0	DM	0	0
	LAMA	33	DM	20	23
	Referred	17	DM	20	37
	Deaths	1	DM	1	1
	Total daily patient count	1245	DM	1251	1316
	Bed occupancy rate	47%	DM	50%	57%
	ALS	3	DM	nan	2
3	Pediatrics				
	Allocated beds	35	DM	35	35
	Admissions	579	DM	622	676
	Disc/DOR not on same day	471	DM	507	498
	Disc/DOR on same day	0	DM	0	0
	LAMA	12	DM	15	14
	Referred	20	DM	22	74
	Deaths	30	DM	29	31
	Total daily patient count	1075	DM	1050	1085
	Bed occupancy rate	100%	DM	100%	100%
	ALS	2	DM	nan	2
4	OB/GYN				
	Allocated beds	33	DM	33	33
	Admissions	531	DM	529	524
	Disc/DOR not on same day	405	DM	377	394
	Disc/DOR on same day	0	DM	0	0
	LAMA	43	DM	55	35
	Referred	14	DM	21	13
	Deaths	0	DM	1	0
	Total daily patient count	445	DM	440	1023
	Bed occupancy rate	47%	DM	100%	100%
	ALS	2.15	DM	nan	2
5	Eye				
	Allocated beds	6	DM	6	6
	Admissions	29	DM	27	21
	Disc/DOR not on same day	26	DM	24	17
	Disc/DOR on same day	0	DM	0	0
	LAMA	1	DM	0	0
	Referred	0	DM	0	0
	Deaths	0	DM	0	0
	Total daily patient count	47	DM	41	29
	Bed occupancy rate	25%	DM	23%	16%
	ALS	2.3	DM	nan	2
6	ENT				
	Allocated beds	5	DM	5	5
	Admissions	11	DM	15	14
	Disc/DOR not on same day	7	DM	12	11
	Disc/DOR on same day	0	DM	0	0
	LAMA	0	DM	0	0
	Referred	0	DM	0	0
	Deaths	0	DM	0	0
	Total daily patient count	25	DM	23	22
	Bed occupancy rate	16%	DM	15%	14%
	ALS	3.5	DM	nan	2
7	Cardiology				
	Allocated beds	25	DM	25	25
	Admissions	182	DM	171	158
	Disc/DOR not on same day	122	DM	120	110
	Disc/DOR on same day	0	DM	0	0
	LAMA	4	DM	5	4
	Referred	18	DM	21	16
	Deaths	22	DM	14	15
	Total daily patient count	407	DM	372	424
	Bed occupancy rate	52%	DM	0	55%
	ALS	2.4	DM	0	0

We could not retrieve data on indoor admissions for September. The occupancy rate was 100% in the pediatric and gynecology ward, >50% in cardiology and surgery, and 70-100% in medicine. The eye and ENT departments had a lower rate of bed occupancy (<50%).

The number of surgical procedures is mentioned in (Table 6).

**Table 6:** Operation theater (OT) records Number of surgical procedures performed DM: Data missing

		August	September	October	November
1	Surgery under general anesthesia	DM	146	DM	DM
2	Surgery under spinal anesthesia	DM	208	DM	DM
3	Surgery under local anesthesia	DM	91	DM	DM
4	Other surgery	DM	7	DM	DM

The list of the main diagnoses that became a reason for admission to medical or surgical wards is given in Tables 7, 8 (Appendices).

## Discussion

Health system data not only furnish crucial information pertaining to healthcare utilization and expenditure but also provide substantial insights into the patterns of various diseases [7]. Health systems exhibit diversity in their performance-based capabilities, which include but are not limited to the provision of essential medications, skilled birth attendance, FP services, antenatal care, and immunization. The importance of management in health systems is well-documented; however, further research is required to determine the precise role of a district as a management unit and health system management at the district level [8]. The goal of the current data analysis is to look into the disease burden that the district healthcare unit is responsible for. This medical facility accommodates patients in its OPDs and offers indoor facilities for surgical procedures. Furthermore, it is equipped with a fully operational laboratory and a system for monitoring communicable diseases. Additionally, it provides FP services, antenatal care, and vaccinations for both the mother and child.

Surveys are undertaken on a global scale to estimate disease burden, identify the most prevalent diseases, and analyze disease epidemiology. For instance, the Asia-Pacific region is susceptible to the development of communicable diseases [9], and South Asia faces challenges from preventable ailments (measles, pneumonia, and diarrhea accounted for two-thirds of the roughly 3.7 million child deaths attributed to such causes in 2000). Central Asia has witnessed a 29% increase in new cases of AIDS, with India having the second-highest prevalence of AIDS and HIV [10]. Asia-Pacific exhibits a higher prevalence of dengue [11]. Over four months, pneumonia was responsible for 14 deaths and diarrhea was responsible for two deaths in our study population.

The global prevalence of hepatitis B is estimated to be 296 million, while hepatitis C has an additional 1.5 million cases [12]. hepatitis B virus (HBV) is more prevalent among patients with liver cirrhosis in Asia and Africa (8-61%) compared to Europe, America, and Oceania (3-41%). The co-occurrence of HBV and hepatitis C virus infections exceeds 50% in countries across Asia and Africa. The primary cause of liver cirrhosis [13] is alcohol consumption, which ranges from 0% to 41% in Oceania, 16-78% in Europe, and 17-52% in America. In 2019, 229 million cases of malaria have been documented on a global scale [14]. A meta-analysis encompassing research conducted between 2006 and 2021 documented a cumulative incidence of malaria amounting to 23.3% (1.68-99.79%) (19). From 1990 to 2019, the age-standardized incidence rate (ASR) decreased by an average of 0.80% annually [15]. According to the WHO, the number of deaths due to malaria reached 409,000 in 2019. Children under five years (67%), pregnant females, and people living in sub-Saharan Africa (94%) and Southeast Asia (3%) were the most vulnerable populations [14]. Among the diseases that need surveillance, 150 cases of viral hepatitis, 155 cases of tuberculosis, and 211 cases of suspected malaria were reported in the present data. No cases of AIDS/HIV or acute flaccid paralysis were reported.

The growth in the burden of non-communicable diseases (NCDs) over the past 10 years has created a barrier to development goals such as poverty reduction, human security, economic stability, and health fairness [16]. Systemic sclerosis incidence and prevalence have increased globally [17], and multiple chronic diseases are more prevalent among women (28.4%) than among men (25.9%), and the risk increases with age [18]. Of the 56.9 million deaths worldwide in 2016, an estimated 40.5 to 2 million were due to NCDs [19]. In 2021, two-thirds of the deaths in Southeast Asia will be associated with NCDs. Half of the deaths occurred between 30 and 69 years of age, and cardiovascular diseases accounted for 3.9 million deaths, along with cancer, chronic respiratory diseases, and diabetes. However, there has been inconsistent progress in risk factor reduction and NCD management [20]. The medicine department of the District Headquarters Hospital, Kotli, Azad Jammu and Kashmir also receives a large bulk of patients with NCDs.

Low- and middle-income nations account for 70-80% of NDC-related mortality. According to WHO NCD Country Profiles 2014, Pakistan has a double burden of NCDs and communicable diseases, with NCDs accounting for approximately half of all deaths. About 1210 homes in Nurpur Shahan were surveyed; 34.4% of people had high blood pressure or IHD. Smoking, drug use, and alcohol addiction are also risk factors [16]. In a survey in Lahore from 2018-2019, 64.5% of the respondents were women. Of the participants, high blood pressure (40.1%), diabetes (15.8%), and IHD (17.0%) were common, and the risk factors were obesity or overweight (68.8%), prehypertension (37.0%), smoking (13.6%), and alcohol use (1.8%). Age was the most important risk factor; 42.4% were between the ages of 30 and 39 years, and 23.8% were in adults aged 60 years and older [21]. High prevalences of peptic ulcer disease, hypertension (HTN), DM, and IHD were reported in the present data. HTN, DM, and IHD cases also needed admission to the medical ward in some instances.

A survey of 10 cities in Pakistan found that approximately 54% of 14,531 children received vaccination, while 14% had received no vaccination. This study highlights the importance of gender equality and access to healthcare [22]. According to an audit conducted in 2018, the most common diseases in Pakistan are typhoid fever, measles, tuberculosis, respiratory infections, diarrhea, and hepatitis A, B, and C. Episodes of dengue, malaria, and chikungunya occur in between. Infections of different organ systems and rabies caused by dog bites are also common [8]. The United Nations Pakistan reported that diarrhea, malaria, and typhoid fever are constantly increasing in Pakistan, with 44000 cases of malaria reported only in 2022 in the southern province [23]. Dengue is a serious issue in Pakistan [24]. The present data also exhibited the same trend. Admissions in the medical ward were for the same diseases as mentioned above, particularly diarrhea and pneumonia in children, tuberculosis, viral hepatitis A and E, and DM-related complications in adults. The dengue cases needed 32 admissions in August, and there were two deaths.

Most of these health conditions are managed by private-sector practitioners, including general physicians and medical specialists [8]. Baseline data analysis and a comprehensive understanding of the burden of communicable and NCDs can help formulate effective policies and plans. Pakistan has a huge burden of communicable diseases, and the number is on the rise. Multiple socioeconomic and demographic factors, such as the lack of a well-developed healthcare system, poverty and illiteracy, population pressure, the burden of internally displaced and external migrants, and a lack of prevention strategies, are all factors that enhance the problem [8]. Initiatives for global health and development must address socioeconomic and health-sector limitations. The management and treatment of communicable diseases should be independently determined by each nation [25]. This study, apart from describing the burden on a healthcare system, also aimed to emphasize the use of DHIS. As mentioned in the methodology section, the DHIS data entry was done manually in the first phase. The details are clearly given in all sections of the DHIS forms for August. But, for September, a section of the data regarding the indoor patient details was missing, which is a sign of a lack of consistency in keeping data. In the second phase, the DHIS dashboard was introduced as an electronic database for healthcare systems. The data for October and November has a lower number of missing values (the data for the number of surgical procedures is missing) as compared to September. This is a sign that a manual data entry system has less compliance, is difficult to use, and is hard to maintain. This analysis encourages the use of electronic databases (DHIS).

Limitations

The DHIS is a novel system in Pakistan. It is actively being used in different health centers in Punjab and other provinces. Kotli is a small district in AJK, Pakistan, and it has recently adopted the DHIS. There are still certain limitations to the data, like a lack of consistency in the process, i.e., consistency in the records updated and delicate details in certain parts of DHIS. There is a lack of interest in maintaining data on the dashboard and the hospital staff probably does not realize the importance of a well-established electronic database (which is visible in the form of missing values in certain areas of DHIS). At this point, DHIS data from district Kotli is insufficient to provide a detailed picture of the burden on the healthcare system and the improvement in providing services over time.

## Conclusions

For nations with constrained healthcare systems and funds, primary health care (PHC) is the only viable approach for managing NCDs. However, PHC systems intended for infectious diseases have not sufficiently adapted to the growing requirement of chronic care for NCD. Research using health information databases offers numerous benefits, such as the evaluation of large data sets and unexpected prevalence of disease in certain populations, such as a higher prevalence of disease in one gender or age group. Health information system-based data analysis or studies are less expensive and faster but lack scientific control over data collection.

## Appendices

**Table 7:** Main diagnosis in patients admitted to the medical ward

Medical ward		August 2023	September 2023	October 2023	November 2023
1	Diarrhea/dysentery (under five years)				
	Total admission	53	0	0	34
	Total deaths	2	0	0	0
2	Diarrhea/dysentery (over five years)				
	Total admission	34	0	0	34
3	Pneumonia (under five years)				
	Total admissions	105	0	0	750
	Total deaths	3	0	0	4
4	Pneumonia (over five years)				
	Total admissions	13	0	0	15
	Total deaths	3	0	0	4
5	Asthma				
	Total admission	4	0	0	30
	Total deaths	1	0	0	0
6	Chronic obstructive airways				
	Total admission	20	0	0	30
	Total deaths	3	0	0	0
7	Pulmonary tuberculosis				
	Total admission	5	0	0	6
	Total deaths	7	0	0	0
8	Typhoid				
	Total admission	11	0	0	0
	Total deaths	3	0	0	0
9	Diabetes mellitus				
	Total admission	38	0	0	44
	Total deaths	1	0	0	1
10	Viral hepatitis A and E				
	Total admission	3	0	0	2
11	Viral hepatitis C				
	Total admission	1	0	0	7
12	Meningitis				
	Total admission	5	0	0	4
	Total deaths	1	0	0	1
13	Chronic liver diseases				
	Total admission	5	0	0	30
	Total deaths	3	0	0	0
14	Chronic renal diseases				
	Total admission	16	0	0	3
15	Dengue fever				
	Total admission	32	0	0	0
	Total deaths	2	0	0	1
Cardiac diseases					
16	Congestive cardiac failure (CCF)				
	Total admission	13	0	0	10
	Total deaths	1	0	0	2
17	Hypertension				
	Total admission	33	0	0	56
	Total deaths	2	0	0	1
18	Ischemic heart disease (IHD)				
	Total admission	33	0	0	35
	Total deaths	5	0	0	2

**Table 8:** Main diagnosis in patients admitted to the surgical ward CVA: Cerebrovascular accident; DNS: Deviated nasal septum; ENT: Ear, nose, throat

	Surgical ward	August 2023	September 2023	October 2023	November 2023
1	Acute appendicitis				
	Total admission	77	0	0	55
2	Burns				
	Total admission	2	0	0	5
3	Cholelithiasis/cholecystitis				
	Total admission	6	0	0	21
4	Hernias				
	Total admission	3	0	0	7
5	Hyperplasia of prostate				
	Total admission	6	0	0	10
6	Urolithiasis				
	Total admission	5	0	0	7
7	Fractures				
	Total admission	23	0	0	15
Eye					
8	Cataract				
	Total admission	23	0	0	77
ENT					
9	DNS				
	Total admission	0	0	0	3
Gynecological					
10	Fibroid uterus				
	Total admission	1	0	0	1
Obstetrics/maternal complication					
11	Antepartum hemorrhage (APH)				
	Total admission	3	0	0	3
12	Complications of abortion				
	Total admission	10	0	0	3
13	Ectopic pregnancies				
	Total admission	4	0	0	3
14	Postpartum hemorrhage (PPH)				
	Total admission	2	0	0	2
15	Pre-eclampsia/eclampsia				
	Total admission	3	0	0	3
16	Puerperal sepsis				
	Total admission	0	0	0	2
Neurological/neurosurgical					
17	CVA/stroke				
	Total admission	52	0	0	85
	Total deaths	5	0	0	6
18	Head injuries				
	Total admission	14	0	0	118
19	Mental disorder				
	Total admission	3	0	0	0
Any other unusual diseases					
20	Miscellaneous				
	Total admission	1657	0	0	183
	Total deaths	48	0	0	70
